# Matrix metalloprotein-triggered, cell penetrating peptide-modified star-shaped nanoparticles for tumor targeting and cancer therapy

**DOI:** 10.1186/s12951-020-00595-5

**Published:** 2020-03-17

**Authors:** Fangyuan Guo, Qiafan Fu, Kang Zhou, Chenghao Jin, Wenchao Wu, Xugang Ji, Qinying Yan, Qingliang Yang, Danjun Wu, Aiqin Li, Gensheng Yang

**Affiliations:** 1 College of Pharmaceutical Science, Zhejiang University of Technology, #18 Chaowang Road, Hangzhou, 310032 People’s Republic Of China; 2 Zhejiang Share Bio-Pharm Co., Ltd, Hangzhou, 310019 China

**Keywords:** Cell-penetrating peptide, Cleavable peptide, Matrix metalloproteinase, Enzyme-responsive nanoparticles, Targeted drug delivery

## Abstract

**Background:**

Specific targeting ability and good cell penetration are two critical requirements of tumor-targeted delivery systems. In the present work, we developed a novel matrix metalloprotein-triggered, cell-penetrating, peptide-modified, star-shaped nanoparticle (NP) based on a functionalized copolymer (MePEG-Peptide-Tri-CL), with the peptide composed of GPLGIAG (matrix metalloprotein-triggered peptide for targeted delivery) and r9 (cell-penetrating peptide for penetration improvement) to enhance its biological specificity and therapeutic effect.

**Results:**

Based on the in vitro release study, a sustained release profile was achieved for curcumin (Cur) release from the Cur-P-NPs at pH 7.4. Furthermore, the release rate of Cur was accelerated in the enzymatic reaction. MTT assay results indicated that the biocompatibility of polymer NPs (P-NPs) was inversely related to the NP concentration, while the efficiency toward tumor cell inhibition was positively related to the Cur-P-NP concentration. In addition, Cur-P-NPs showed higher fluorescence intensity than Cur-NPs in tumor cells, indicating improved penetration of tumor cells. An in vivo biodistribution study further demonstrated that Cur-P-NPs exhibited stronger targeting to A549 xenografts than to normal tissue. Furthermore, the strongest tumor growth inhibition (76.95%) was observed in Cur-P-NP-treated A549 tumor xenograft nude mice, with slight pulmonary toxicity.

**Conclusion:**

All results demonstrated that Cur-P-NP is a promising drug delivery system that possesses specific enzyme responsiveness for use in anti-tumor therapy.

## Background

Targeted selection and cellular uptake of drugs are major challenges in successful cancer chemotherapy. For conventional chemotherapy, non-specific tissue biodistribution and low cellular uptake contribute to limited therapeutic effects and cause severe side effects in normal tissues [1, 2]. Therefore, a novel, safe, and efficient delivery system with tumor cell-specific targeting and efficient cellular uptake properties is highly needed. Over the past few decades, nanomedicines have displayed great promise in improving aqueous solubility of lipophilic drugs, altering drug metabolism and biodistribution (EPR effect), and reducing side effects during cancer chemotherapy [3, 4]. However, the elimination of nanoparticles (NPs) from systemic circulation (by the reticuloendothelial system) and the unspecific cellular uptake of the NP carrier have greatly limited drug bioavailability in vivo. Thus, precise surface engineering of NPs with specific ligands, which can improve their targeting ability, cellular penetration, and circulation longevity, is required.

Matrix metalloproteinases (MMPs), one class of abundant proteases surrounding tumors that is involved in tumor progression, tumor angiogenesis, and metastasis [5, 6], are overexpressed throughout the extracellular matrix of most cancer cells compared to the normal cell environment [7, 8]. MMPs could therefore be recognized as targets of enzyme-triggered therapeutics [9]. To date, a variety of homing peptides [10–16] that exhibit MMP action have been developed and have demonstrated encouraging results. Frustratingly, not all NPs arriving at tumor sites are effectively internalized by tumor cells [17, 18]. Therefore, a drug delivery system with good internalization capability is required. Cell-penetrating peptides (CPPs), which are composed of natural amino acids (positive lysine and arginine residues), are known to promote cell penetration to deliver cargo [19]. Therefore, the conjugation of targeting ligands with a CPP could be a potential strategy to achieve cancer cell selectivity.

**Fig. 1:**
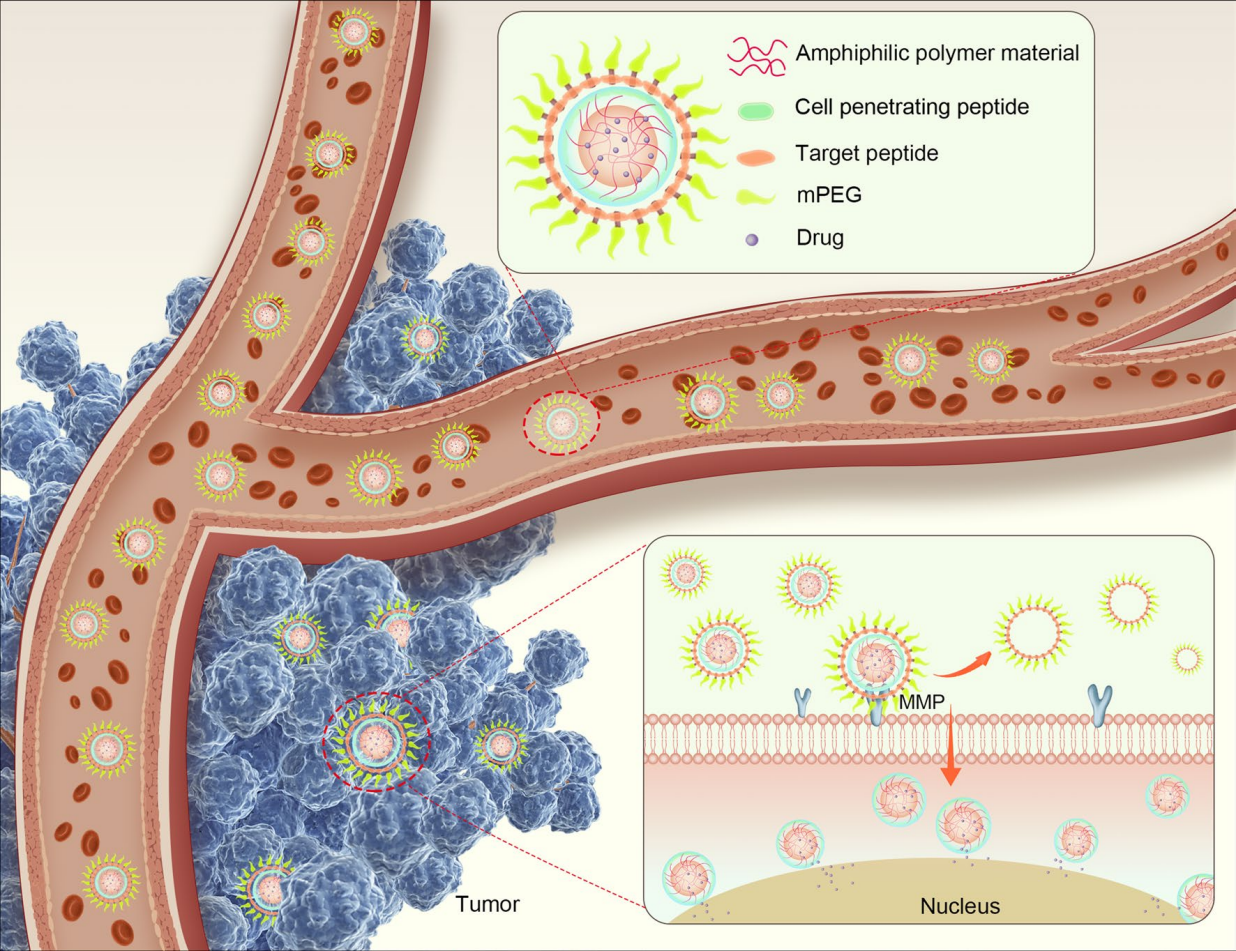
Detailed schematic diagram of MePEG-Peptide-Tri-CL for cancer treatment

In this study, we selected curcumin (Cur) for use as a model drug owing to its exceptional anti-cancer activity, safety profile, and lack of ability to induce multidrug resistance. However, its poor water solubility, poor absorption, and rapid clearance have greatly inhibited its clinical application [20]. To overcome these disadvantages of Cur, we aimed to develop an enzyme-triggered CPP-mediated NP (MePEG-Peptide-Tri-CL) to improve cancer chemotherapy using Cur. In MePEG-Peptide-Tri-CL, the hydrophobic segment, Tri-CL, contains the loaded drug, and the peptide moieties of GPLGIAG and r9 have the merits of selective targeting and enhanced cellular uptake, respectively. The star-shaped Tri-CL displays a higher drug-loading capacity than straight-chain polymers, and the short peptide, GPLGIAG, is present as a linker that is cleavable by MMP proteases [21]; the exposed r9 has been used as a CPP to enhance the cellular uptake of NPs [21, 22]. Polyethylene glycol (PEG) chains can protect CPP from proteolysis and rapid renal and/or liver clearance and prolong the half-life in blood plasma; a detailed schematic diagram is displayed in Fig. 1. In addition, in vitro assays and in vivo studies were performed to evaluate the cytotoxicity of the biomaterials as well as the anticancer activities and the selective targeting behavior of the developed formulations.

## Materials and methods

### Materials

(ACP)-GPLGIAGQr9-(ACP) was purchased from ChinaPeptides Co. Ltd. (Shanghai, China). Poloxamer-188 was from BASF (Shanghai, China). 4-Aminophenylmercuric acetate (APMA), collagenase IV, stannous 2-ethylhexanoate [Sn(Oct)_2_], and MePEG (Mw = 1.9 kDa) were received from Sigma-Aldrich (Shanghai, China). Fetal calf serum, pancreatic enzymes, and Dulbecco’s modified Eagle’s medium (DMEM) were from Hangzhou Yu Jie Biotechnology Co. Ltd. (Hangzhou, China). Curcumin was purchased from Hangzhou Guang Lin Biological Pharmaceutical Co. Ltd. (Hangzhou, China). Dialysis bags (MWCO = 14 kDa) were obtained from Gene Star Co. (Shanghai, China). Other reagents (analytical or chromatographic grade) were obtained from Aladdin Chemicals (Shanghai, China).

### Synthesis of enzyme-responsive star-shaped copolymer

**Fig. 2:**
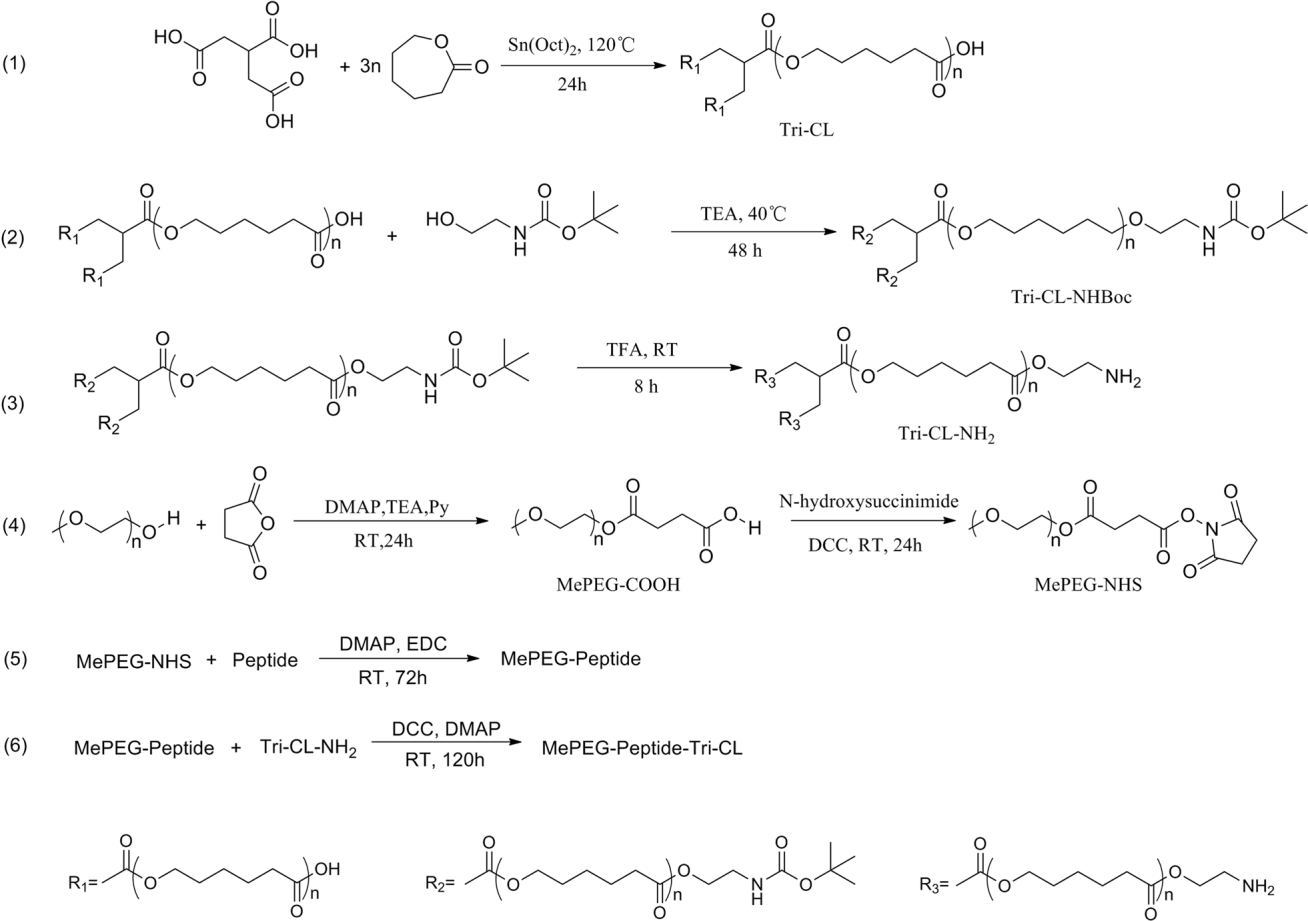
Reaction scheme for MePEG-Peptide-Tri-CL preparation

MePEG-Peptide-Tri-CL was prepared via a multistep synthetic process with MePEG, tricarballylic acid, ε-caprolactone, and peptide as the initiators. The detailed process is presented in Fig. 2.

### Synthesis of Tri-CL-NH_2_

Tricarballylic acid (0.1765 g, 1 mmol) and ε-caprolactone (3.4245 g, 30 mmol) were melted in a 100-mL three-neck flask at 120 °C. Sn(Oct)_2_ (catalyst, 12 μL, 0.37 mmol) was then added, and the solution was mixed to create a homogeneous solution. The polymerization reaction was performed at 120 °C under dry nitrogen for 24 h. Tri-CL was purified by precipitation from dichloromethane in diethyl ether (1/10, v/v), and dried *in vacuo* at 40 °C for 24 h.

Tri-CL (1.0530 g, 0.1 mmol) was dissolved in methylbenzene (10 mL), followed by the addition of trimethylamine (94 μL, 0.68 mmol) and 2-(tert-butoxycarbonylamino)-1-ethanol (0.0645 g, 0.4 mmol); the mixture was then stirred for 48 h under dry nitrogen. The Tri-CL-NHBoc crude product was added dropwise to cold methanol, and the precipitate was obtained following centrifugation at 6000 rpm for 30 min and drying *in vacuo* at 25 °C for 24 h.

Tri-CL-NHBoc (0.6576 g, 0.06 mmol) was dissolved in a mixed solution of dichloromethane (DCM, 10.0 mL) and trifluoroacetic acid (0.0274 g, 0.24 mmol), stirred for 8 h at 25 °C, followed by simultaneous washing of the reaction solution with saturated KHCO_3_ solution and distilled water. The extraction process was repeated three times, and the DCM solution was collected and dehydrated using MgSO_4_. The Tri-CL-NH_2_ product was purified by precipitation in cold methanol (1:15, v/v) isolated by filtration and vacuum drying at 25 °C for 24 h.

### Synthesis of MePEG-NHS

MePEG (Mw = 1900 Da, 7.6 g, 4 mmol), butanedioic anhydride (0.8 g, 8 mmol), 4-dimethylaminopyridine (DMAP, 73.3 mg, 0.6 mmol), and triethylamine (556 μL, 4 mmol) were fully dissolved in pyridine (60 mL), and the solution was stirred under dry nitrogen at room temperature for 24 h until the reaction was complete. After evaporation, the crude product of MePEG-COOH was precipitated from DCM (20 mL) in cold ethyl ether (1:15, v/v). The precipitate was dried *in vacuo* at 25 °C for 48 h.

MePEG-COOH (3.0000 g, 1.5 mmol) and *N*-hydroxy succinimide (0.6906 g, 6 mmol) were transferred to a 50-mL three-neck flask and fully dissolved in acetonitrile (15 mL). Thereafter, N,N′-dicyclohexylcarbodiimide (DCC, 1.0316 g, 5 mmol) was added, the mixture was stirred under an atmosphere of N_2_ at room temperature for 24 h before purification, using the same process used for MePEG-COOH.

### Synthesis of MePEG-Peptide-Tri-CL

Peptide (50 mg, 0.0213 mmol), 1-ethyl-3-(3-dimethylaminopropyl)carbodiimide hydrochloride (EDC, 27.3 mg, 0.142 mmol), and DMAP (17.4 mg, 142 mmol) were dissolved in acetonitrile–water solution (20/80, 10 mL), and stirred under dry nitrogen for 2 h in an ice-water bath to activate the peptide. MePEG-NHS (49.9 mg, 0.0178 mmol) dissolved in acetonitrile (2 mL) was added, and the reaction was stored at room temperature for 72 h. Thereafter, the MePEG-Peptide crude product was obtained by lyophilization.

MePEG-Peptide (138.3 mg, 0.0229 mmol), DCC (35.7 mg, 0.1718 mmol), and DMAP (21.2 mg, 0.1718 mmol) were dissolved in DCM (15 mL), stirred under dry nitrogen in an ice-water bath for 2 h, followed by addition of Tri-CL-NH_2_ (92.0 mg, 0.0057 mmol). The mixture was further stirred at 25 °C for 96 h until the reaction was complete. MePEG-Peptide-Tri-CL purification was carried out by dialysis (MWCO = 14 kDa), followed by lyophilization.

^1^H-NMR and FT-IR spectra of MePEG-Peptide-Tri-CL were recorded on a Bruker AVANCE III spectrometer and Nicolet 6700 spectrometer, respectively. The number average molecular weight (Mn), weight-average molecular weight (Mw), and polydispersity index (PDI) of the polymers in each reaction equation were analyzed by gel permeation chromatography (GPC) using an LC-20AT instrument with an RID-10A detector [23].

### Preparation of Cur-loaded NPs

To prepare Cur-loaded NPs, Cur (3.2 mg) and MePEG-Peptide-Tri-CL (64.0 mg) were co-dissolved in 4 mL acetone, while Poloxamer-188 (40.0 mg) was dissolved in 20 mL of distilled water to form an aqueous phase. Thereafter, the lipid phase was added drop-wise to the aqueous phase under magnetic stirring. The mixture was stirred for 0.5 h and dried *in vacuo* for 1 h to remove acetone. The obtained primary NP suspension (Cur-P-NPs) was filtered through a 0.45-μm membrane to remove free Cur and achieve a homogeneous suspension.

### Characterization of Cur-loaded NPs

Characterization particle size, PDI, and zeta potential of Cur-P-NPs was performed using a laser particle analyzer (Malvern Zetasizer Nano-ZS90; Malvern, UK). For morphological analysis, Cur-P-NPs were negatively stained with 2 wt% sodium phosphotungstate before analysis by transmission electron microscopy (TEM) using JEOL JEM-1010 at 15,000 × magnification.

The Cur-P-NP drug content was determined by ultraviolet (UV) spectrophotometry with a detection wavelength of 420 nm. Cur-P-NPs were centrifuged at 19,000 rpm for 30 min. The precipitate was collected and lyophilized. Drug entrapment efficiency (EE) and drug loading (DL) were calculated by using the following equations:

1 $$\text{EE}\%=\frac{\text{Weight}\,\text{of}\,\text{drug}\,\text{in}\, \text{NPs}}{\text{Weight} \,\text{of}\,\text{feed}\,\text{drug}}\times 100$$

2 $$\text{DL}\%=\frac{\text{Weight}\,\text{of}\,\text{drug}\,\text{in}\, \text{NPs}}{\text{Weight} \,\text{of}\,\text{NPs}}\times 100$$

Additionally, to further study improvements in the water solubility of Cur in Cur-P-NPs, the maximum content of Cur in 0.1 M PBS (pH 7.4) and in Cur-P-NPs was measured using the method described above.

### In vitro stability of Cur-P-NPs

Cur-P-NP (1 mL) and DMEM [6 mL, containing 10% fetal bovine serum (FBS) complete medium] were co-incubated at 37 °C for 24 h. Then, at 1, 6, 12, 24, and 48 h, 1 mL of the sample solution was collected, and the particle diameter and PDI of Cur-P-NPs were measured. The test was repeated three times, and the data were expressed as the mean ± standard deviation.

### In vitro drug release

To evaluate the effect of MMP-triggered drug release, Cur-P-NPs (the control group) and Cur-P-NPs with collagenase IV (containing MMP-2/9; treatment group) were prepared. The Cur release rate was examined in PBS by dialysis at 37 °C and pH 7.4. Briefly, collagenase IV was activated at 37 °C with a 2.5 mM APMA solution [24]. Cur-P-NPs solution was mixed with activated collagenase IV to obtain Cur-P-NPs (+ 50 μg/mL collagenase IV). Cur-P-NPs (5 mL) and Cur-P-NPs (+ 50 μg/mL Collagenase IV, 5 mL), with the same Cur content (150 μg/mL), were each dialyzed (MWCO = 14 kDa) against 50 mL PBS in an incubator, with shaking at 100 rpm. At predetermined time points (0–216 h), 3.0 mL of external solution was removed and replaced with an equivalent volume of fresh buffer. Free Cur was determined by ultraviolet spectrophotometry. All experiments were carried out in triplicates.

### Measurement of MMP levels

L929 (mouse embryonic fibroblasts) and A549 (human lung carcinoma) cells were seeded in 96-well plates at a density of 1 × 10^5^ cells/well and incubated with DMEM with 10% (v/v) FBS at 37 °C in 5% CO_2_/air and 100% relative humidity for 24 h. Thereafter, the culture solution was centrifuged at 1,000 rpm for 20 min, and 50 μL of supernatant was removed for measurement of the MMP-2/MMP-9 levels in L929 and A549 cells using ELISA kits.

### In vitro cytotoxicity study

The toxicity of free P-NPs in L929 cells was measured using the 3-(4,5-dimethylthiazol-2-yl)-2,5-diphenyltetrazolium bromide (MTT) assay. Cells were seeded at 1 × 10^5^ cells/well in 96-well plates, 24 h before treatment. A 100-μL volume with different contents of sterilized free P-NPs or medium only (negative control, 100% cell viability) solutions were added to cells and incubated for another 48 h. All samples were prepared in triplicates. A 20-μL volume of MTT labeling reagent was then added to each well and incubated with the cells for 4 h at 37 °C. Cell viability was determined using a microplate reader by measuring the absorbance at 570 nm, using the formula below:

3 $$\text{Cell viability}\left(\text{\%}\right)=\frac{{[\text{A}]}_{\text{test}}}{{[\text{A}]}_{\text{control}}} \times 100\text{\%}$$

where: [A]_test_ and [A]_control_ represent the absorbance values of the test and negative control solutions, respectively.

### In vitro anti-proliferation efficacy

A549 (human lung carcinoma) was used as the model cell line to assess the efficacy of the Cur-P-NP solution against lung cancer cell viability by MTT analysis. Cells were seeded in 96-well plates at a density of 1 × 10^5^ cells/well and incubated with 100 μL of different diluted Cur-P-NPs suspensions or medium only (negative control, 100% cell viability) for 48 h. The residual operation and calculation were performed in the same manner as described in section "In vitro cytotoxicity study". Additionally, the IC_50_ of each sample was calculated by SPSS.

### Cellular uptake

The enhancement effect of Cur-P-NPs via cellular uptake was confirmed by fluorescence microscopy (20 × magnification) and confocal laser scanning microscopy (CLSM) with A549 cells. Fluorescence microscopy: A 100-μL volume of Cur-DMSO [DMSO/H_2_O = 1/1000 (v/v)] aqueous solution (control group), free P-NPs, Cur-NPs (preparation using MePEG-Tri-CL without peptide modification), and Cur-P-NPs was co-cultured with A549 cells at a Cur concentration of 50 μg/mL at 37 °C for 4 h in 96-well plates (10^5^ cells/well). Before detection by fluorescence microscopy, the medium was discarded, and the cells were washed thrice with PBS. CLSM: Cur-DMSO, Cur-NPs, and Cur-P-NPs were incubated with A549 cells at a Cur concentration of 50 μg/mL for 3 h in 24-well plates (10^5^ cells/well) at 37 °C. After incubation, the medium was removed, and the cells were washed three times with PBS. DAPI counterstaining was performed for 20 min. The cells were then washed three times, and the coverslips were removed from the plates and sealed onto slides with Fluorescent Mounting Medium. Visualization was then performed by CLSM.

To further investigate the function of the peptide in the internalization of Cur-P-NPs, A549 cells were pre-incubated in DMEM in 6-well plates (10^5^ cells/well) containing 10 μM 1,10-phenanthroline monohydrate (used as an MMP inhibitor) for 2 h. After washing the cells in PBS, they were treated with Cur-P-NPs or Cur-NPs containing 50 μg/mL Cur for 1 h. Thereafter, the medium was removed, and the cells were washed twice with PBS prior to analysis by flow cytometry. Additionally, these steps were repeated for the control group (A549 cells without treatment with 10 μM 1,10-phenanthroline monohydrate). All experiments were carried out in triplicates.

### Animal studies

Male BALB/c nude mice (age, 5–6 weeks; weight, 20 ± 4 g) were obtained from Zhejiang Academy of Medical Sciences (Hangzhou, China) for use in the study. All experimental procedures were conducted in conformity with institutional guidelines for the care and use of laboratory animals at Zhejiang University of Technology, Hangzhou, China, and the National Institutes of Health Guide for Care and Use of Laboratory Animals (Publication No. 85–23, revised 1996). The lung xenograft tumor model was developed by subcutaneous injection of 1 × 10^7^ A549 cells (0.2 mL) into each left fore of nude mice. Once the tumor was formed (~ 100 mm^3^), features such as biodistribution and pharmacodynamics were evaluated.

### Biodistribution studies

A 0.2-mL volume of Cur-DMSO, Cur–NPs, or Cur-P-NPs at a Cur content of 50 μg/mL was injected into tumor-bearing nude male mice via the tail vein. For imaging studies, nude male mice were anesthetized at pre-established time points (1 h, 6 h, and 24 h), and images were obtained with a small animal imager (IVIS, Lumina XRMS III) at wavelengths of 488 nm and 520 nm for excitation and emission, respectively. After 24 h, the nude mice were sacrificed, and major organs including the heart, liver, spleen, lungs, kidneys, and tumors were harvested. The fluorescence intensity of these organs was also examined using the small animal imager.

To evaluate the concentration of Cur in the main tissues, 36 (6 × 6 groups) tumor-bearing nude male mice injected with the same dose of Cur-DMSO, Cur-NPs, or Cur-P-NPs were sacrificed at 1 h or 24 h. The heart, liver, spleen, lungs, kidneys, and tumors were harvested and then weighed and homogenized using a tissue homogenizer following the addition of 200 μL of PBS. Cur was extracted from tissue homogenates using acetonitrile. After sonication for 5 min and centrifugation for 10 min at 8,000 rpm, the supernatant was dried by nitrogen flow, and another 50 μL of acetonitrile was added to re-dissolve the sediment. Cur content was then measured by HPLC.

### In vivo pharmacodynamics

Tumor-bearing nude mice were randomly divided into 6 groups (n = 6/group). Each group of mice was then treated every 2 days via injection in the tail vein (0.2 mL) with physiological saline (negative control), or a 0.8 mg/kg dose of Cur-DMSO, Cur-NPs, or Cur-P-NPs. The tumor volume was measured every two days, and the tumor volume and tumor growth inhibition were calculated using the following formulas:

4 $$\text{Tumor volume}=\frac{\text{a}*{\text b}^{2}}{2}$$

5 $$\text{Tumor growth inhibition}={\frac{(\text{V}_{\text{c}}-\text{V})}{\text{V}_\text{c}}}$$

where a and b represent the length and width of the tumor, respectively, and where Vc and V represent the tumor volume for the control group and sample group, respectively.

Additionally, the weight of nude mice was also measured every two days during the experimental period (15 days).

### Histological analysis

Fifteen days post-injection, the tumor-bearing nude mice were sacrificed. The heart, liver, spleen, lungs, kidneys, and tumors were removed and washed with PBS and then fixed in 4% formaldehyde for histological examination with hematoxylin and eosin (H&E) to assess the in vivo biocompatibility of the formulations.

### Statistical analysis

Results are expressed as the mean ± standard error. Differences between groups were examined for statistical significance using Student’s t-test. P-values < 0.05 were considered statistically significant.

## Results and discussion

### Enzyme-responsive star-shaped copolymer characterization

**Fig. 3.**
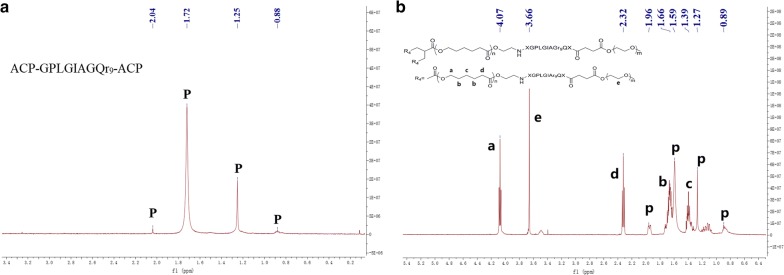
^1^H-NMR spectra of peptide (**a**) and MePEG-Peptide-Tri-CL (**b**)

**Fig. 4:**
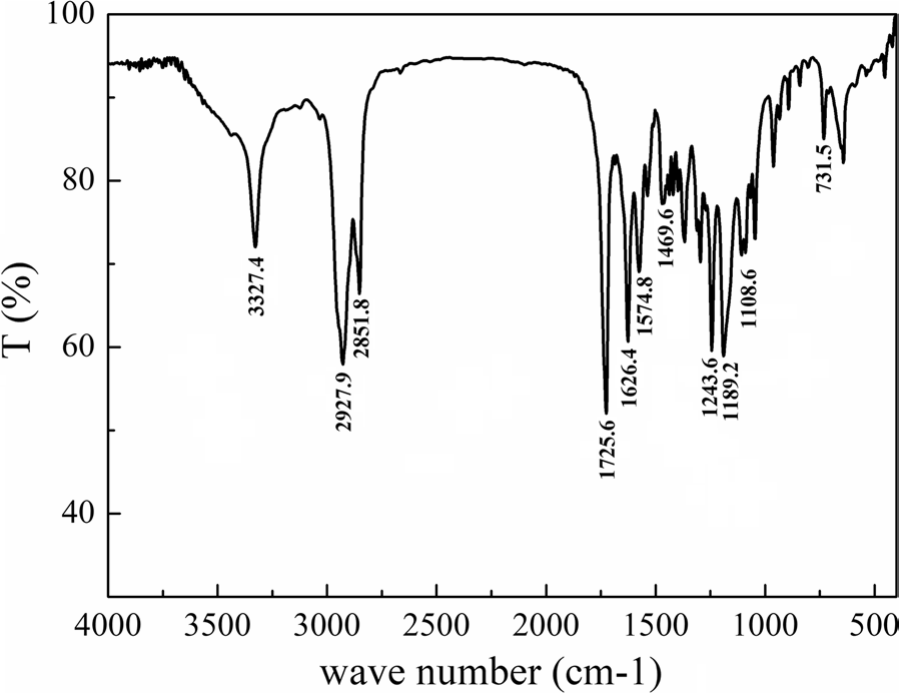
FT-IR spectra of MePEG-Peptide-Tri-CL

In the ^1^H-NMR spectrum of (ACP)-GPLGIAGQr9-(ACP) (Fig. 3a), the characteristic peaks of the peptide are shown at 2.04, 1.72, 1.25, and 0.88 ppm, while different moieties are shown in the ^1^H-NMR spectrum of MePEG-Peptide-Tri-CL (Fig. 3b). When compared, distinct chemical shifts of the peptide (p) could be labeled. For the MePEG moiety, the methylene group in the backbone was located at 3.66 ppm (e); for the Tri-CL moiety, the peaks displayed at 4.07 (a), 2.32 (d), 1.67 (b), and 1.40 ppm (c) represented the different protons of methylene. The FT-IR spectrum of MePEG-Peptide-Tri-CL is illustrated in Fig. 4, with peaks at 3327.4, 1626.4, and 1574.8 cm^−1^ corresponding to the vibrations of O–H, C = O, and N–H bonds in the peptide, respectively. The peak at 1108.6 cm^−1^ is characteristic of C–O–C bond stretching and attributed to MePEG. Additionally, the signals at 2851.8, 1469.6, and 731.5 cm^−1^ reflect the C–H vibrations of the methylene groups of MePEG and Tri-CL. All the above signals demonstrated the successful synthesis of MePEG-Peptide-Tri-CL.

Mn, Mw, and PDI of the prepolymers measured by GPC are listed in Table 1. The molecular weight of each polymer was close to that of the target. Moreover, the narrow distribution observed for the polymers is beneficial for the subsequent formation of iso-dispersed NPs.

**Table 1:** Mn, Mw, and PDI of the prepolymers

	Mn (g·mol^−1^)	Mw (g·mol^−1^)	PDI
Tri-CL	9591	10,530	1.09
Tri-CL-NH_2_	10,024	12,129	1.21
MePEG-NHS	2398	2611	1.09
MePEG-Peptide	5908	6037	1.02
MePEG-Peptide-Tri-CL	15,894	18862	1.18

### Characterization of NPs

**Fig. 5:**
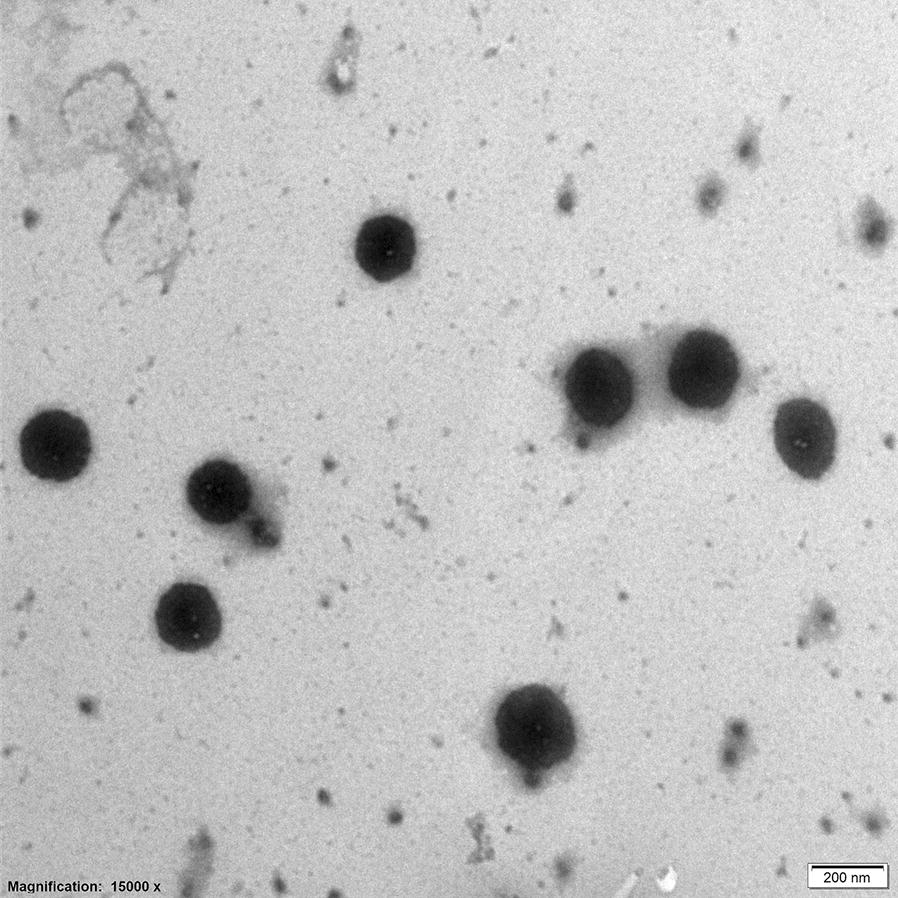
TEM photograph of Cur-P-NPs (40,000 × magnification)

As shown in the electron micrographs presented in Fig. 5, Cur-P-NPs were almost uniform, with a spherical shape and approximately 200 nm in size. The average diameters of Cur-P-NP and free P-NP were 215 and 184 nm, respectively, with relatively narrow PDIs of 0.141 and 0.139, respectively (Table 2). When compared, a slight increase in particle size and PDI was observed after Cur loading into NPs, indicating high stability during NP preparation. The zeta potential exhibited a positive charge, ranging from 4.53 to 5.90 mV; this was due to the positively-charged arginine in the r9 peptides. Moreover, the positively charged corona could improve the efficacy of cellular uptake. The EE% and DL% values of Cur-P-NPs were 82.48% and 7.11%, respectively. Additionally, the Cur contents in 0.1 M PBS and Cur-P-NPs were 0.634 and 235 μg/mL, respectively. Thus, the Cur concentration in Cur-P-NPs was 371 times higher than that in PBS, suggesting that the water solubility of Cur was greatly improved after encapsulation of Cur into the NPs.

**Table 2:** Physicochemical characteristics of Cur-P-NPs

Sample	Particle size (nm)	PDI	Zeta potential (mV)	EE%	DL%
Cur-P-NPs	215.0 ± 6.183	0.141 ± 0.013	5.90 ± 0.424	82.48	7.11
free P-NPs	184.0 ± 2.548	0.139 ± 0.019	4.53 ± 0.512	-	-

### In vitro stability of Cur-P-NPs

**Fig. 6:**
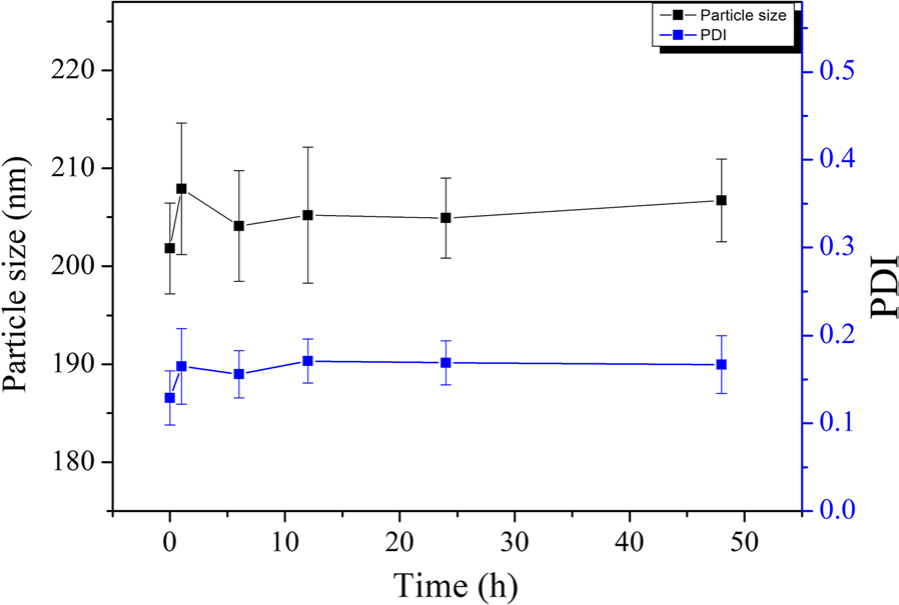
In vitro stability of Cur-P-NPs in DMEM with 10% FBS

Stability of the Cur-P-NP nanocarrier in the blood is important for efficient drug delivery. To simulate the blood environment, we incubated cells in DMEM with 10% FBS with Cur-P-NPs at 37 °C for 48 h. The results shown in Fig. 6 demonstrate that Cur-P-NPs exhibit excellent stability based on particle size and PDI measurements.

### In vitro drug release

**Fig. 7:**
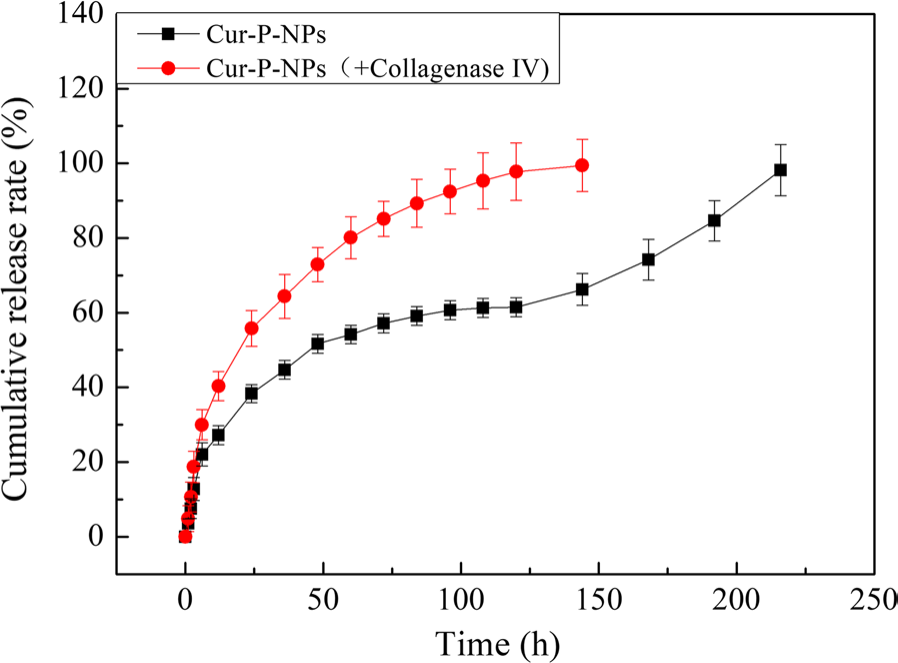
Accumulative release of Cur in vitro at pH 7.4

Kinetic curves representing Cur release from Cur-P-NPs and Cur-P-NPs (+ 50 μg/mL Collagenase IV) are shown in Fig. 7. By comparing these release curves, a near overlap could be seen in the first 3 h, with a cumulative release rate ranging from 13.85% to 16.78%. This rate is due to the fast diffusion of Cur on either the surface or superficial layers of the Cur-P-NP delivery systems. From 6–144 h, the inner Cur (a hydrophobic drug) was released by dissolution and diffusion. In this period, Cur-P-NPs (+ 50 μg/mL Collagenase IV) were fully released and displayed a faster Cur release rate than Cur-P-NPs. This result suggests that internal diffusion channels were formed in the Cur-P-NP delivery system by an enzyme shear reaction, which further accelerated the release of Cur. From 144–216 h, the total cumulative release of Cur reached 98.18% for Cur-P-NPs, demonstrating an accelerated release. This indicates that copolymer degradation and NP disintegration could accelerate drug diffusion.

### Measurement of MMP levels

The MMP-2 and MMP-9 levels of L929 cells were 0.0011 and 0.0014 ng/mL (n = 10^5^), respectively, and the corresponding levels in A549 cells were 0.0465 and 0.1564 ng/mL (n = 10^5^), respectively. Thus, the MMP-2 concentration in A549 tumor cells was 42.3 times higher and the MMP-9 concentration was 117 times higher than the corresponding levels in normal L929 cells, confirming that A549 is an MMP-positive cell line.

### In vitro cytotoxicity study

**Fig. 8:**
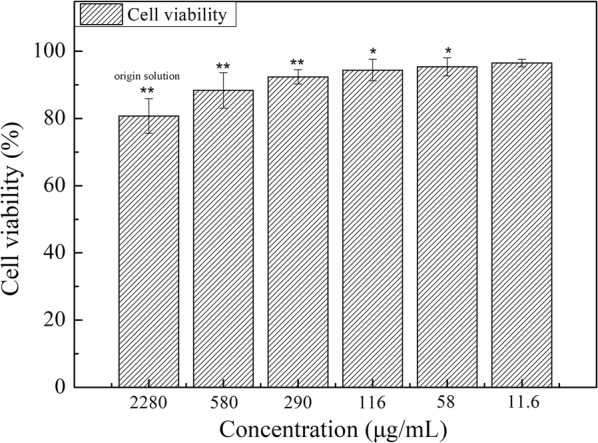
The cell viabilities of blank-P-NPs against L929 (**P < 0.01, *P < 0.05 compared to culture media)

The results of the MTT viability assays are given in Fig. 8. Cell viability increased as the P-Tri-CL content decreased, indicating that free P-Tri-CL NPs displayed dose-dependent cytotoxicity. In addition, total cell viability ranged from 80.76% to 96.51%, indicating that the particles possessed good biocompatibility.

### In vitro anti-proliferation efficacy

**Fig. 9:**
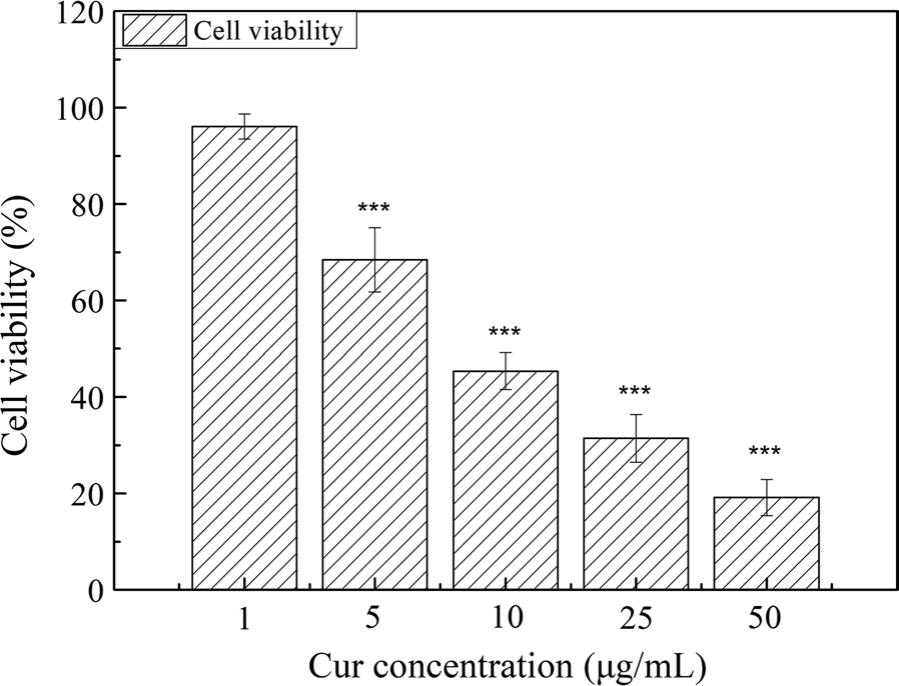
The anti-proliferation effects of Cur-P-NPs against A549 cells (***P < 0.001, **P < 0.01, *P < 0.05 compared to culture media)

The A549 cell line was used to investigate the in vitro anticancer efficacy of Cur-P-NPs at Cur concentrations from 1 to 50 μg/mL using an MTT cell viability assay. Correspondingly, P-NP concentrations ranging from 11.60 to 580 μg/mL (Fig. 9) were deemed safe for cells (according to the in vitro cytotoxicity study). Moreover, cell viability decreased as Cur content increased, indicating that the inhibited growth of A549 cells by Cur-P-NPs was proportional to Cur concentration. After treatment with 1 μg/mL Cur-P-NPs, there was no evident anticancer activity; however, when co-cultured with equivalent concentrations of 5, 10, 25, and 50 μg/mL, good anticancer efficacy was achieved, with cell viabilities of 96.05%, 68.43%, 45.37%, 31.44%, and 19.17%, respectively. Therefore, to achieve effective treatment, the Cur concentration should be higher than 5 μg/mL. Indeed, the IC_50_ value of Cur-P-NPs against A549 cells was determined to be 10.123 μg/mL.

### Cellular uptake

**Fig. 10:**
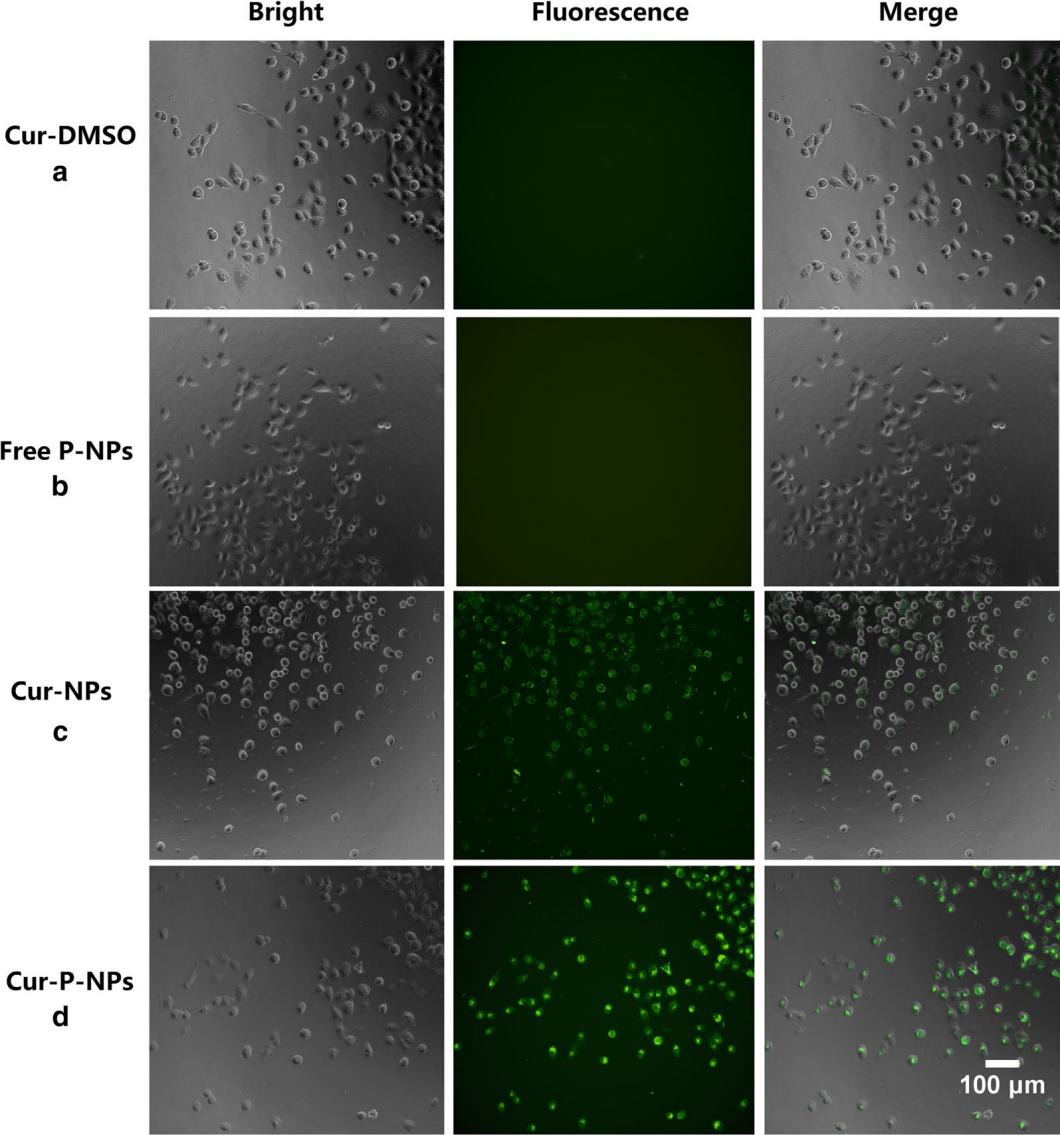
Fluorescence microscopy images of cellular uptake of **a** Cur-DMSO, **b** Free P-NPs, **c** Cur-NPs, and **d** Cur-P-NPs in A549 cells (20 × magnification)

**Fig. 11:**
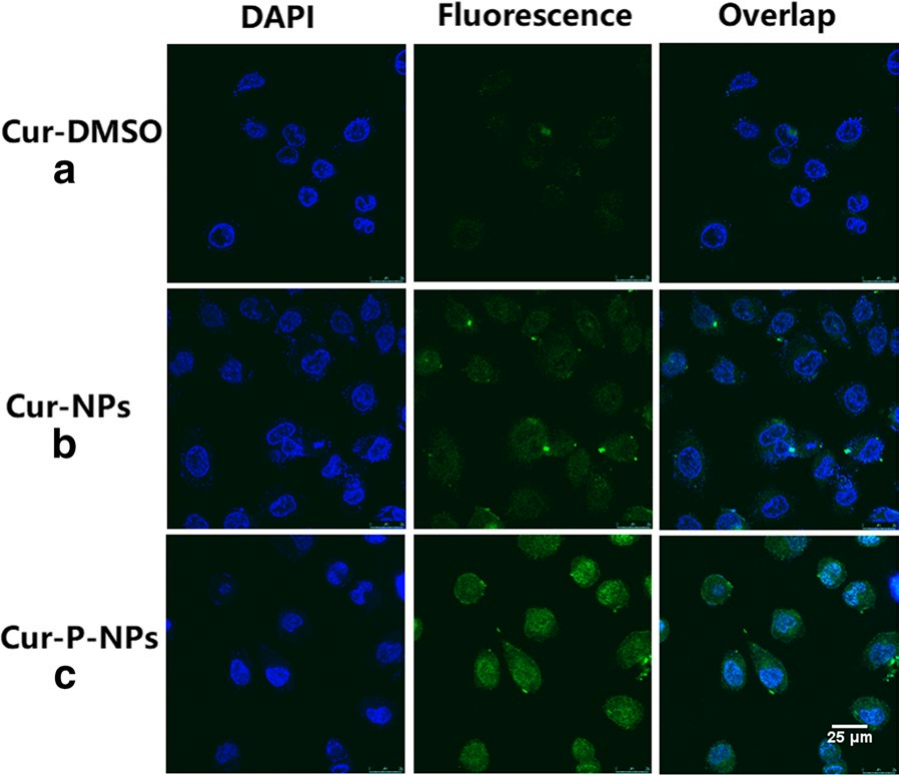
CLSM images of cellular uptake of **a** Cur-DMSO, **b** Cur-NPs, and **c** Cur-P-NPs in A549 cells. Blue indicates cell nuclei, and green indicates Cur

As Cur exhibits fluorescence, the differences in cellular uptake by A549 cells for Cur-DMSO (negative control), free-P-NPs, Cur-NPs, and Cur-P-NPs were assessed using fluorescence microscopy (Fig. 10). Free-P-NPs (Fig. 10b) showed little fluorescence, demonstrating that the total fluorescence effect was induced by Cur. As shown in Fig. 10a, c, and d, an evidently weaker fluorescence intensity was observed for Cur-DMSO compared to Cur-NPs and Cur-P-NPs, indicating enhanced cellular uptake efficiencies because of the NPs [23]. Moreover, a stronger fluorescence intensity of Cur-P-NPs (Fig. 10d) was observed when compared to that of Cur-NPs (Fig. 10c), suggesting that GPLGIAG (cleavable peptide) and r9 (CPP) exerted a synergistic effect on the internalization of Cur-P-NPs [25]. Interestingly, PEGylation of polymers can hinder cellular uptake efficiency [26, 27]; however, in the present study, Cur-P-NPs showed excellent uptake ability. A reasonable inference is that the ‘MePEG-GPLGIAGQ-r9′ might be cleaved to ‘MePEG-GPLGIAGQ’ and ‘r9-NPs’ by the MMP enzyme, leaving r9-NPs, and enabling a tremendous improvement in its uptake ability [28]. The fluorescence images detected by CLSM are shown in Fig. 11. These results were the same as those obtained by fluorescence microscopy. The intensity of the fluorescence effect was in the following order: Cur-P-NPs > Cur-NPs > Cur-DMSO. In addition, Cur-DMSO and Cur-NPs displayed weak fluorescence intensities in cell nuclei (Fig. 11a, b), while Cur-P-NPs (Fig. 11c) displayed strong fluorescence. This indicates that the latter has a stronger cellular uptake ability and higher cytotoxicity [29].

**Fig. 12:**
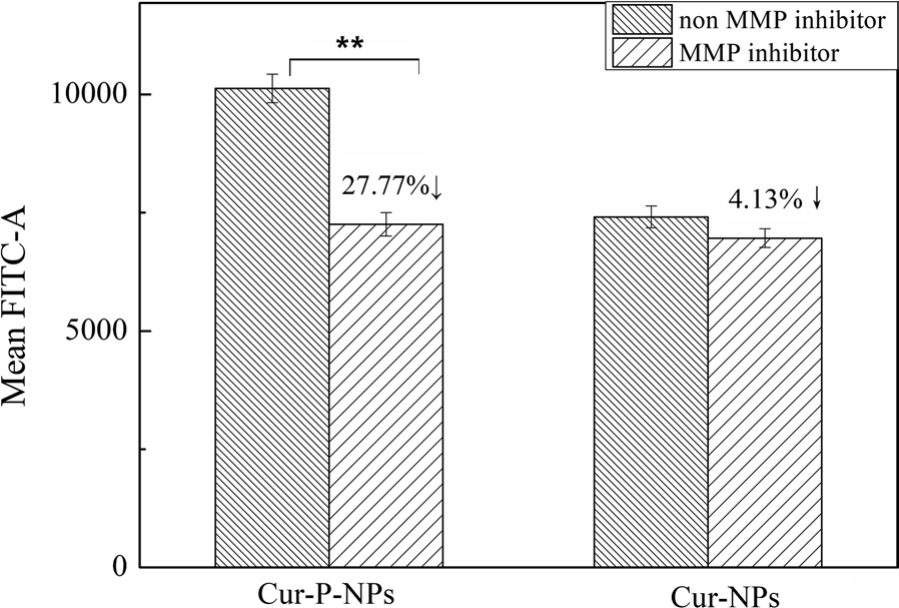
Flow cytometry analysis of A549 cells incubated with Cur-P-NPs and Cur-NPs in the absence and presence of an MMP inhibitor (**P < 0.01)

Furthermore, 1,10-phenanthroline monohydrate was used as an MMP inhibitor to study the function of the peptide in the ability of the nano-vectors to internalize [30–32]. The results of quantitative flow cytometry are presented in Fig. 12. For Cur-P-NPs (non MMP inhibitor) and Cur-NPs (non MMP inhibitor), more than 30% of the mean of fluorescein isothiocyanate (FITC) values were measured to increase in Cur-P-NPs than that of Cur-NPs. Meanwhile, A549 cells pretreated with MMP inhibitor could downregulate the level of MMP. Compared to the internalization ability of Cur-P-NPs in the A549 cells with different MMP levels, approximately 27.77% of the mean FITC values were found to decrease in A549 cells incubated with the MMP inhibitor. This result highlights that a lack of MMP could cause a decrease in the cleavage ability. In addition, less r9 was exposed on the surface of the NPs, decreasing cellular uptake. For Cur-NPs, due to the presence of the non-targeting peptide in the nanocarrier, only a 4.13% mean FITC value decrease was measured in pretreated A549 cells.

### Biodistribution studies in vivo

**Fig. 13:**
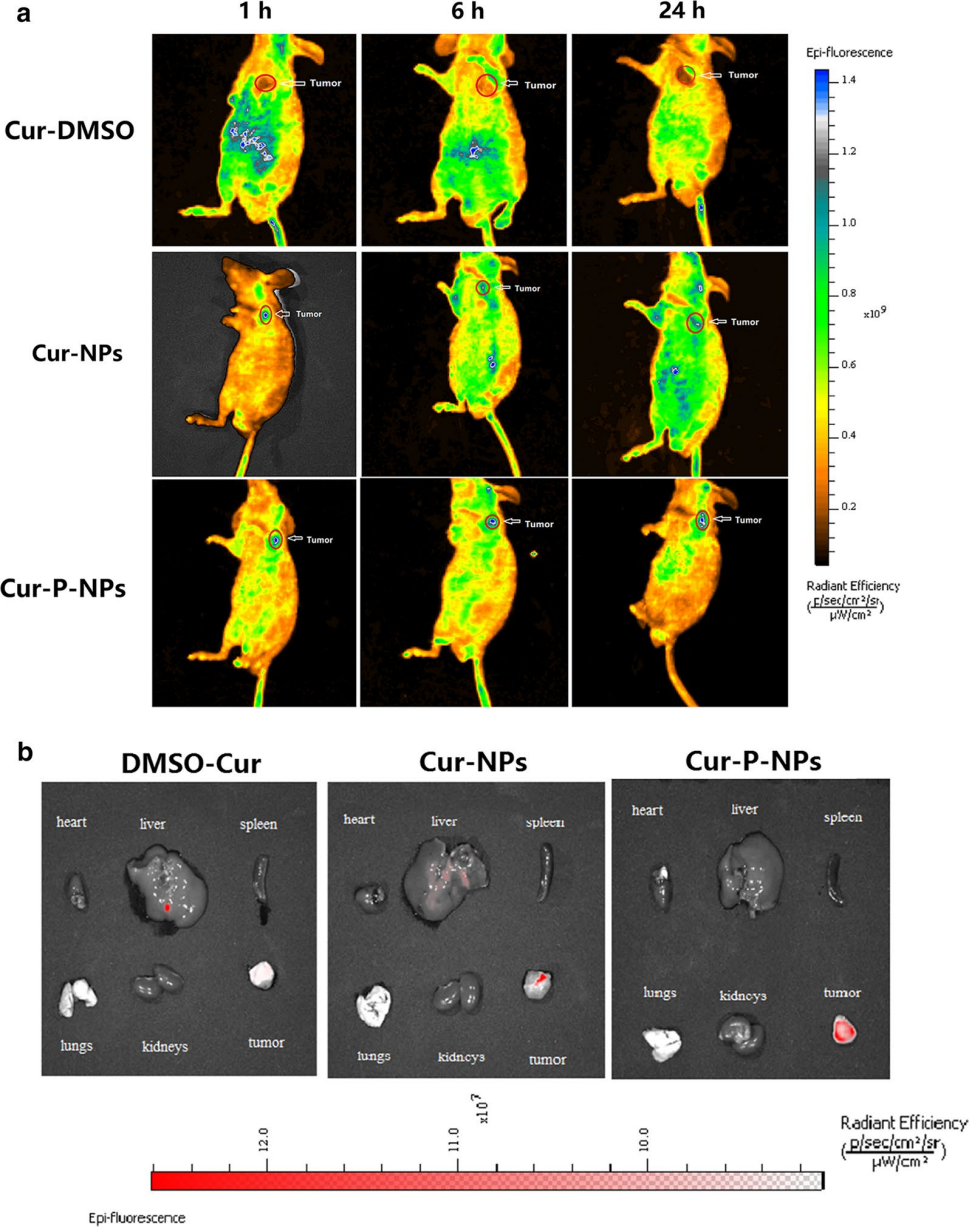
Biodistribution imaging of **a** the whole body and **b** main organs after i.v. injection of Cur

In vivo imaging was employed to evaluate the targeting efficiency of Cur-DMSO, Cur-NPs and Cur-P-NPs in A549 xenograft-bearing nude mice. The major organs (heart, liver, spleen, lungs, kidneys) and tumors at 24 h post-injection were excised for ex vivo imaging to analyze the distribution in tissues. As displayed in Fig. 13a, b, mice treated with Cur-DMSO had a small amount of tumor accumulation following intravenous injection via the tail vein at 24 h. Cur content was mainly distributed in the abdomen during the first hour; thereafter, the fluorescence effect of Cur decreased with time and almost vanished at 24 h due to metabolism. This further demonstrates, by ex vivo tissue imaging, that Cur residues were not evident in the major organs (except the liver). For Cur-NPs, a visible fluorescence effect was observed in the tumor xenografts 1 h after injection, while a small amount of Cur was detected in the major organs. Therefore, owing to the EPR effect, a well-targeted distribution within a short time was observed. Six hours or 12 h after injection, the Cur concentration was significantly elevated in other tissues, suggesting that the tumor-targeting effect of Cur-NPs had decreased. Because the presence of MePEG may limit the permeability of Cur-NPs, this may have resulted in the NPs being prone to be captured and metabolized by other organs, which is consistent with the results from ex vivo tissue images. In contrast to Cur-NPs, Cur-P-NPs displayed the highest fluorescence effect at the tumor site, while less fluorescence was observed in other organs throughout the 24-h period. Meanwhile, a stronger tumor-targeting ability by Cur-P-NPs was also confirmed by ex vivo tissue imaging. Besides the EPR effect (passive targeting), this is commonly caused by active targeting. MePEG-GPLGIAGQ and r9-NPs were cleaved in a reaction catalyzed by MMP [33]; this contributed to the enhanced cellular uptake and decreased capture and metabolization rate by other organs.

**Fig. 14:**
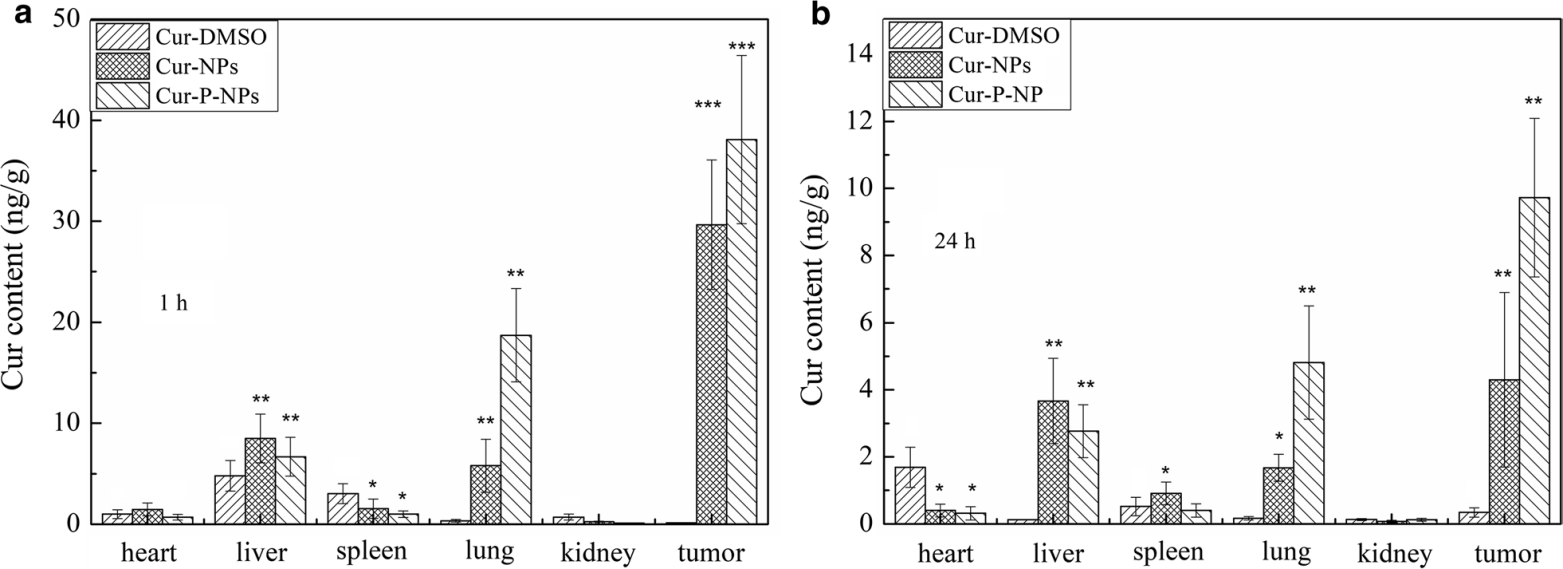
Cur content in major organs and tumor **a** 1 h, **b** 24 h (***P < 0.001, **P < 0.01, *P < 0.05 compared to Cur-DMSO)

The results of the Cur quantitation in major organs and the tumor of xenograft-bearing nude mice at 1 h and 24 h are shown in Fig. 14. After 1 h of treatment (Fig. 14a), Cur-DMSO was mainly present in the liver and spleen, while no significant uptake was observed in other tissues due to the metabolic process used by the reticuloendothelial system (RES). The Cur content decreased to 0.139 ng/g tumor, highlighting poor bioavailability of Cur-DMSO. Cur-NPs and Cur-P-NPs resulted in a significant increase in Cur content in the tumor compared to the organs, with Cur contents of 26.524 and 38.490 ng/g tumor, respectively. Liver and lungs were other organs displaying Cur-NP and Cur-P-NP accumulation, with Cur contents of 6.267 and 4.292 ng/g, respectively, in the liver, and 4.476 and 15.633 ng/g lung, respectively, in the lungs. Compared to Cur-NPs, Cur-P-NPs caused a higher Cur content in the MMP-overexpressing A549 tumors; this could be attributed to the function of the GPLGIAGQr9 polypeptide. Based on passive targeting, more Cur-P-NPs were localized at the tumor site owing to the detachment of MePEG and the targeting GPLGIAGQ, which are degraded by MMPs. Furthermore, r9 was exposed on the surface of NPs, prompting cellular uptake (with an estimated 42.6% improvement). In addition, more Cur-P-NPs accumulated in the lung, which is the target organ of lung cancer, indicating that Cur-P-NPs could provide better therapeutic effects against lung tumors than against tumors in other tissues. Therefore, Cur-P-NPs showed the strongest targeting and uptake ability in tumor tissue and lung. After 24 h of treatment (Fig. 13b), a gradual reduction in Cur accumulation in the main tissues and tumor was clearly observed. However, for Cur-NPs and Cur-P-NPs, a high amount of Cur in tumor tissue was still observed, with Cur contents of 4.448 and 10.380 ng/g, respectively.

### In vivo pharmacodynamics

**Fig. 15:**
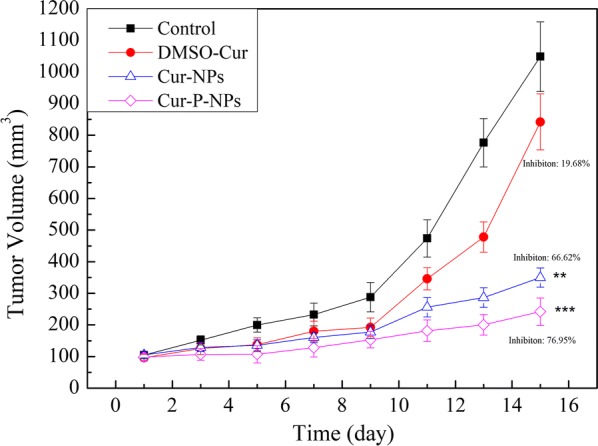
Antitumor activity of Cur in different formulations in A549 tumor-bearing mice (***P < 0.001, **P < 0.01, *P < 0.05 compared to control group)

To investigate the therapeutic effect of Cur in vivo, tumor-bearing nude mice were injected with Cur in different formulations, and the tumor volume and tumor growth inhibition were monitored (Fig. 15). For the control group, the variation in tumor volume displayed a two-phase process: a gradual increment phase (0–9 days) and a burst phase (10–15 days). At the end of the experiment, the tumor volume was 10.1-fold greater than the initial volume, and a similar trend for the tumor volume was observed for the Cur-DMSO group. Due to poor cellular uptake and distribution in vivo, the tumor volume of the mice did not significantly decrease when compared to the control group, where the tumor volume grew to 842.25 ± 88.25 mm^3^ and final tumor growth inhibition was only 19.68%. As expected, due to the EPR effect (passive targeting), treatment with NPs could slow tumor growth (especially in the second phase), which demonstrated the importance of targeted therapies. For the Cur-NP group and Cur-P-NP group, tumor growth inhibition was 66.62% and 76.95%, respectively. Strikingly, Cur-P-NPs displayed a much more efficient tumor growth inhibition, which aligns with the results of cellular uptake and distribution in vivo.

**Fig. 16:**
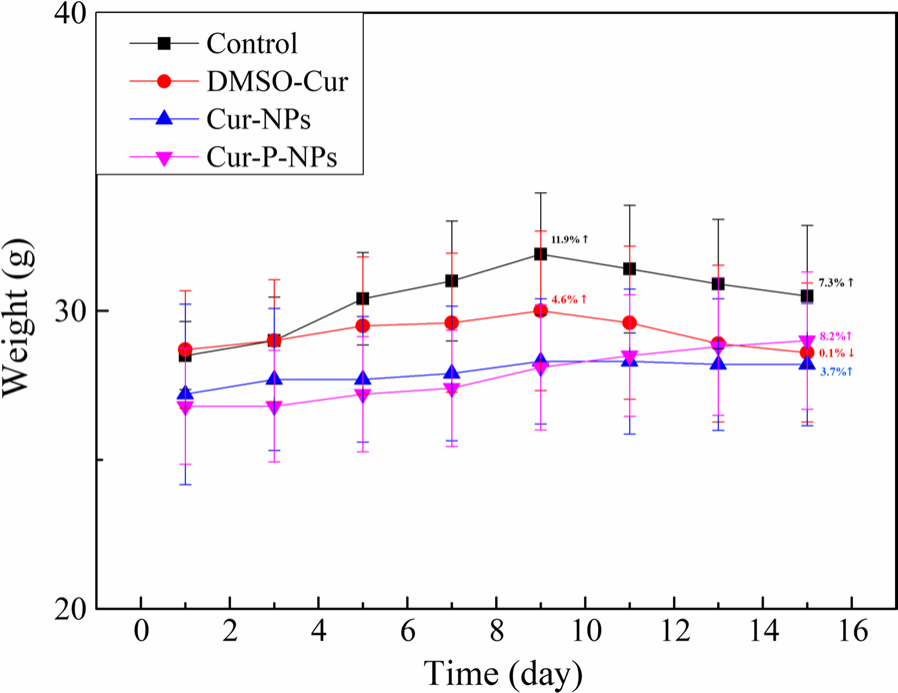
Body-weight change after treatment with Cur in different formulations

Notably, the body weights of the mice closely correlated with the safety evaluation in vivo [34]. As shown in Fig. 16, the control group and Cur-DMSO group displayed decreases in body weight from day 9; the total body weight decrease was 4.6% and 4.7% (compared to day 9) for the control group and Cur-DMSO group, respectively. On the other hand, mice treated with Cur-NPs and Cur-P-NPs experienced slight changes and stable growth in body weight, with a total body weight increase of 3.7% and 8.2% (compared to the initial weight) respectively, suggesting a low systemic toxicity of these samples.

### Histological analysis

**Fig. 17:**
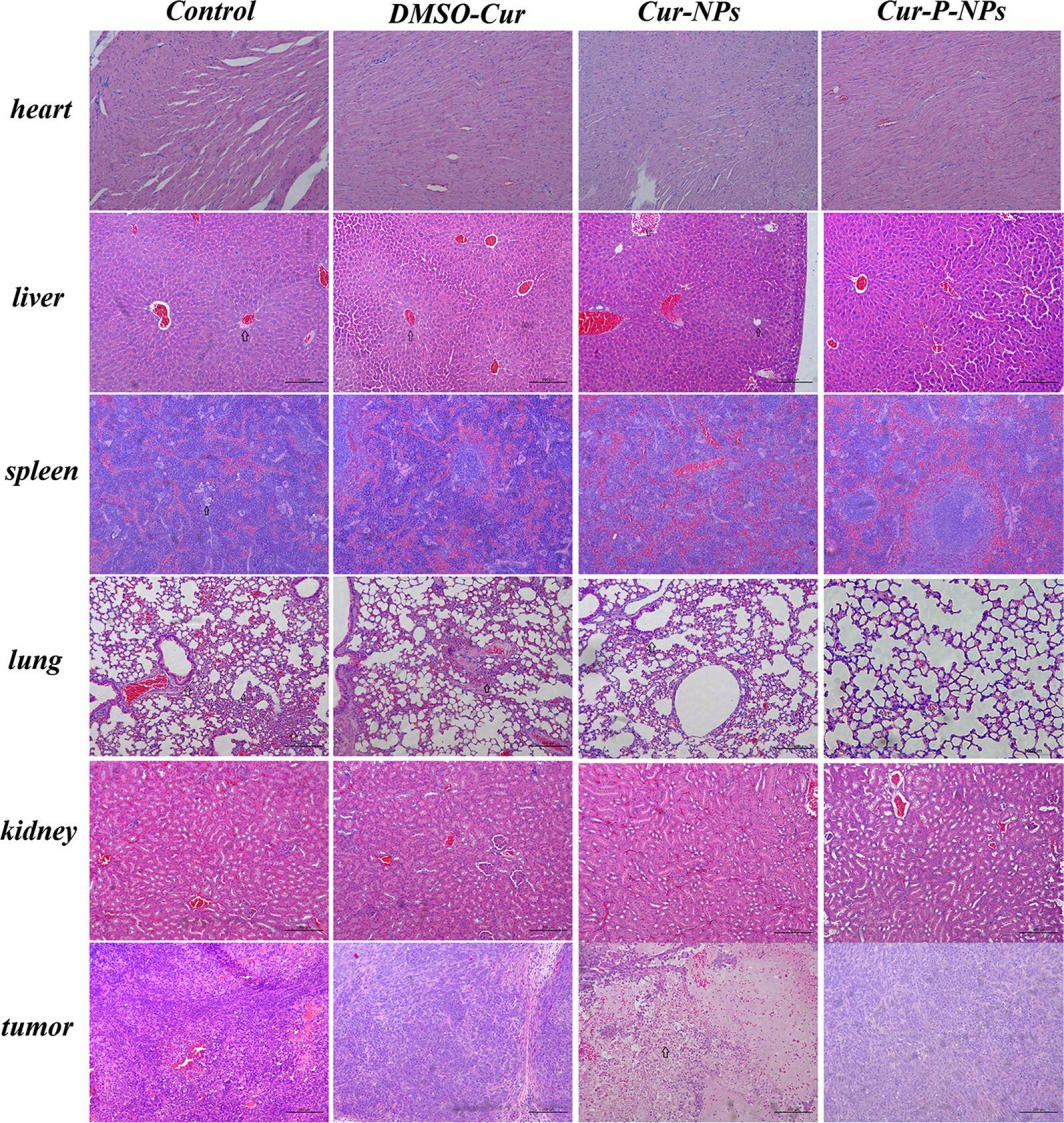
Histological results showing in vivo biocompatibility

To evaluate toxicity in vivo, we performed H&E staining of the heart, liver, spleen, lung, kidney, and tumor tissue after therapy (Fig. 17). For the control group, there was no appreciable abnormality or evident organ damage in the heart, kidney, and tumor tissues. Liver, spleen, and lung tissues, however, displayed local necrosis, atrophy, and inflammatory cell infiltration, respectively. For all treatment groups, spleen cell atrophy disappeared, and both Cur-NPs and Cur-P-NPs were demonstrated to cause tumor tissue necrosis. Concurrently, inflammatory cell infiltration of the liver and lung tissue still existed after treatment with Cur-NPs. In contrast, besides moderate pulmonary toxicity, no appreciable abnormality was observed after treatment with Cur-P-NPs. Consequently, Cur-P-NPs efficiently enhanced the therapeutic index without resulting in any evident side effects.

## Conclusions

In this study, novel enzyme-responsive, cell-penetrating, peptide-modified, star-shaped NPs (Cur-P-NPs) were designed and prepared, with Cur as the targeted drug for delivery to tumor cells. These synthesized NPs displayed enhanced anticancer therapy, reduced side effects, and sustained release profiles in vitro at pH 7.4. The Cur-P-NPs showed stronger penetration of A549 cells than Cur-NPs and Cur-DMSO. In addition, Cur-P-NPs had stronger targeting to A549 xenografts than to normal tissue, demonstrating strong tumor growth inhibition (up to 76.95%). Besides moderate pulmonary toxicity, almost no abnormalities were identified through histological analysis. All results demonstrated that Cur-P-NP is a promising drug delivery carrier with a specific enzyme-responsive ability for use in anti-tumor therapy.

## Abbreviations

(ACP)-GPLGIAGQrrrrrrrrr-(ACP):
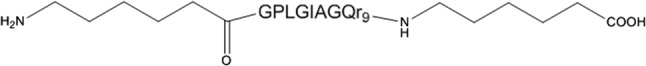


G Gly, P Pro, L Leu, I Ile, A Ala, Q Gln, r D-Arg.
